# Caveats of chronic exogenous corticosterone treatments in adolescent rats and effects on anxiety-like and depressive behavior and hypothalamic-pituitary-adrenal (HPA) axis function

**DOI:** 10.1186/2045-5380-1-4

**Published:** 2011-09-27

**Authors:** Patti Waters, Cheryl M McCormick

**Affiliations:** 1 Department of Psychology and Centre for Neuroscience, Brock University, St Catharines, Ontario, Canada

## Abstract

**Background:**

Administration of exogenous corticosterone is an effective preclinical model of depression, but its use has involved primarily adult rodents. Using two different procedures of administration drawn from the literature, we explored the possibility of exogenous corticosterone models in adolescence, a time of heightened risk for mood disorders in humans.

**Methods:**

In experiment 1, rats were injected with 40 mg/kg corticosterone or vehicle from postnatal days 30 to 45 and compared with no injection controls on behavior in the elevated plus maze (EPM) and the forced swim test (FST). Experiment 2 consisted of three treatments administered to rats from postnatal days 30 to 45 or as adults (days 70 to 85): either corticosterone (400 μg/ml) administered in the drinking water along with 2.5% ethanol, 2.5% ethanol or water only. In addition to testing on EPM, blood samples after the FST were obtained to measure plasma corticosterone. Analysis of variance (ANOVA) and alpha level of *P *< 0.05 were used to determine statistical significance.

**Results:**

In experiment 1, corticosterone treatment of adolescent rats increased anxiety in the EPM and decreased immobility in the FST compared to no injection control rats. However, vehicle injected rats were similar to corticosterone injected rats, suggesting that adolescent rats may be highly vulnerable to stress of injection. In experiment 2, the intake of treated water, and thus doses delivered, differed for adolescents and adults, but there were no effects of treatment on behavior in the EPM or FST. Rats that had ingested corticosterone had reduced corticosterone release after the FST. Ethanol vehicle also affected corticosterone release compared to those ingesting water only, but differently for adolescents than for adults.

**Conclusions:**

The results indicate that several challenges must be overcome before the exogenous corticosterone model can be used effectively in adolescents.

## Background

The World Health Organization has found mood disorders such as anxiety and depression to be a major contributor to disability and loss of years of health in women and in men [1]. Stressful experiences are implicated in the pathogenesis of mood disorders, and dysregulation of the hypothalamic-pituitary-adrenal (HPA) axis is a key feature of depression in particular (reviewed in [2-6]). Clinical research indicates that adolescence is a time of increased risk for mood disorders (reviewed in [7,8]). Changes in the reactivity of the stress systems during times of biological transitions, such as those occurring in the nervous system and gonadal systems during adolescence, are proposed to underlie the increased vulnerability [9].

The use of animal models, though not without limitations, is recognized as an important approach to understanding mood disorders [10,11]. Animal models of depression based on environmental stress (repeated stress exposures administered by the experimenter) have high face and construct validity [12]. Until recently, however, the focus has been on stressors in perinatal life or in adulthood rather than in adolescence [13]. Limitations of many animal models of environmental stress include the lack of control over individual differences and variation in procedures from laboratory to laboratory, which lead to inconsistencies in the literature (reviewed in [14,15]). These limitations may be especially relevant in investigations of adolescent rats, given the sensitivity of gonadal maturation to environmental factors, the ongoing development of the HPA axis, and the relatively short time frame in which adolescent development occurs [13]. One classification system for adolescence (a transitional period with no clear 'onset' or 'offset') involves three stages, a prepubescence/early adolescence period from postnatal days 21 (when rats are typically weaned in the laboratory) to 34, a mid-adolescence period from postnatal days 34 to 46 (time in which most rats first exhibit the physical markers of puberty, such as vaginal opening and balanopreputial separation), and a late adolescence period from postnatal days 46 to 59 [16-18].

The effects of chronic or repeated stress in humans and in animal models are mediated primarily by the prolonged elevations of glucocorticoids (cortisol in humans, corticosterone in rodents) that are the endpoint of activation of the HPA axis [19,20]. Thus, repeated administration of exogenous corticosterone has been proposed as an effective means of circumventing some of the limitations of animal models of chronic stress exposures (reviewed in [14]). Although there is much evidence to indicate that repeated treatment with corticosterone produces reliable changes in a variety of depressive-like behaviors (reviewed in [14]), this approach has yet to be explored in adolescence. One exception is a study of a low dose of corticosterone (20 mg/ml) administered in the drinking water for 2 months (early adolescence into adulthood), which was found to decrease depression-like behavior in male mice [21]. Thus, more studies are required to assess the use of exogenous corticosterone as an animal model using higher doses and within a timeframe limited to adolescence.

Our laboratory has used a social instability stress model in rats to investigate the consequences of exposure to stressors in mid-adolescence (daily 1 h isolation and change of cage partner from postnatal days 30 to 45) (reviewed in [22]). Although social instability stress in adolescence produces lasting changes in neurogenesis and decrements in performance of hippocampal-dependent tasks (for example, [23,24]), effects on anxiety-like and depressive behavior have been modest [25,26], perhaps because of habituation of corticosterone release to the repeated stress procedures in male rats [27]. Thus, in the present experiments, we investigated the effects of repeated administration of exogenous corticosterone over the same timeframe as our adolescent social instability procedure on anxiety-like and depressive behavior. In experiment 1, we used a 40 mg/kg injection of corticosterone and in experiment 2 we used a 400 mg/ml dose administered in the drinking water; both doses and administration procedures have been used extensively with adult rats (for example, injection [28-30] and in water [31-33]). Vehicle and no treatment controls were included in both experiments, and an adult treatment comparison group was included in experiment 2. In both experiments, 24 h after the last treatment day, anxiety-like behavior was evaluated using the elevated plus maze and after another 24 h, depressive behavior was evaluated using the forced swim test, both well validated measures (reviewed in [34,35]). In experiment 2, blood samples were obtained at timepoints after the end of the forced swim test to evaluate treatment effects on stress-induced corticosterone release. The aim of the experiments was to assess the potential of exogenous corticosterone as a model for adolescent mood disorders. The main hypothesis of both experiments was that chronic treatment with exogenous corticosterone in adolescence would increase anxiety-like and depressive behavior in rats.

## Methods

### Experiment 1

#### Animals

Male Long-Evans rats (n = 24) were obtained from Charles River (St Constant, Quebec, Canada) at 22 days of age. Rats were housed in pairs in polycarbonate cages and were provided with a plastic tube in each cage for enrichment throughout the experiment. Rats were identified by tail coloring with a felt tip marker. The rats were kept on a 12 h light, 12 h dark light cycle, and were given unlimited access to rat chow and water. All experimental procedures were consistent with National Institutes of Health Guide for Care and Use of Laboratory Animals (Publication No. 85-23, revised 1985), and Canadian Council on Animal Care guidelines and were approved by the Brock University Institutional Animal Care and Use Committee.

#### Treatment

Starting on postnatal day 30, rats were randomly assigned to one of three treatment conditions. The CORT group (n = 8) was given a daily injection subcutaneously for 16 days of 40 mg/kg/ml corticosterone (Steraloids, Newport, RI, USA) (procedure as in [36,37]) suspended in isotonic saline and 2% Tween 80 (Sigma-Aldrich, St Louis, MO, USA). The VEHIC group (n = 8) was injected daily with vehicle for 16 days. After injection, the rat was returned to the home cage, and each pair of rats that shared a cage was administered the same treatment. Previous research has found this dose of corticosterone injection to produce plasma corticosterone levels of approximately 2,100 ng/ml 1-4 h after the injection [37]. The rats were weighed once every 3 days, and the injection volume was adjusted accordingly. The NO-INJ (no injection, n = 8) control group was not disturbed except for cage maintenance and was weighed only on postnatal days 30 and 45 (first and last injection days). Rats were tested in the elevated plus maze on postnatal day 46 and in the forced swim test on postnatal day 47.

#### Elevated plus maze

The elevated plus maze (EPM), first described by Pellow and colleagues [38], is a well validated measure of anxiety in rodents [34]. The EPM relies on conflict between the propensity of rodents to explore novel territory and their fear of open and elevated areas. The EPM consisted of four arms 50 cm in length, and walls enclosing two arms (closed arms) 42 cm in height constructed out of grey painted plywood. The sides of the open arms had 1.3 cm high ledges. The maze was raised 79 cm off the ground and was kept in a separate room from the colony. During testing, the maze was illuminated with dim white light and white noise (approximately 60 dB) was played. Each rat was placed in the closed arm at the end farthest from the center and was allowed to explore the maze for 5 min. All sessions were videotaped, and tapes were later scored while blind to treatment condition. Tapes were scored for time spent in the open arm (with more time on the open arm, higher percent time on open arm, and higher percentage of open arm entries all indicative of less anxiety) and center hub as well as the total number of entries into closed arms (used as an index of locomotor activity), rears (when the rat stands on its hind legs) and head dips (when the rat reaches its head over the edge of the open arm). Rears and dips are considered measures of exploratory behavior that are exhibited more when less anxious.

#### Forced swim test

The forced swim test (FST) is a measure of behavioral despair (for example, [39]) and consisted of a clear cylinder 20.3 cm in diameter filled 24.1 cm high with water such that a rat was unable to touch the bottom or climb over the side. The water was warmed to 26°C, and it was changed for each rat. Rats were placed in the water and left in the tank for 15 min in experiment 1 and 20 min in experiment 2 (longer time in experiment 2 was used for maximal corticosterone release). All sessions were videotaped, and tapes were later scored while blind to treatment condition. Behavioral measures for analysis were derived from the literature (for example, [40,41]) and included the following four behaviors: swimming (quick movements of forelimbs and/or hindlimbs, including swimming in circles and pedaling), climbing (in a vertical position, pawing at the side of the cylinder with front paws and leveraging the body slightly out of the water), diving (head under water, swimming to the bottom of cylinder, including circling at the bottom) and immobility (reduced movement including floating and slow circling or pedaling). Diving occurred with very low frequency, and was thus not included in statistical analyses. Climbing reflects the most vigorous attempt at escape whereas immobility is the measure considered to represent depressive-like behavior.

Classically the FST is administered in two sessions that are 24 h apart, with latency to immobility and time spent immobile measured in the second session and the first session considered the means to induce a depressive state (for example, [39]). However, stressors or chronic manipulations are found to produce depression-like states and thus differences among groups can be found in the first session (for example, [28,36,42]). Further, the effects of chronic corticosterone treatment for 21 days in adults were the same whether tested with a 1 day version of the test or a 2 day version [29]. Therefore, the experiments described here used a one-session test.

### Experiment 2

#### Animals, treatment, and procedure

Male Long-Evans rats were obtained from Charles River at 22 days of age (n = 20) or at 64 days of age (n = 20) and were housed in age-matched pairs as in experiment 1. Starting on postnatal day 30 or 70, the drinking water was replaced for the CORT-ETOH rats (n = 8 per age group) with a solution of 400 μg/ml corticosterone (Steraloids) dissolved in 2.5% ethanol. To ensure that any effects observed were the result of corticosterone treatment and not ethanol exposure, a second group was given 2.5% ethanol to drink (ETOH rats, n = 6 per group). The WATER rats (n = 6 per group) continued to receive tap water. Each solution was colored with a different food coloring to be easily distinguished from one another. After 16 days, on postnatal day 46 or 86, treatment was removed and tap water was provided to all. During treatment, the mass of water consumed was measured every 2 days. Rats were weighed before and after the 16 days of treatment, but were not handled during the course of treatment, except for cage cleaning thrice weekly. Using weight at the start and end of treatment, the dose of ethanol and of corticosterone consumed by the pair of rats in a cage over a 2 day period was calculated. To calculate the doses of ethanol and corticosterone, the density of each solution was determined by weighing 1 ml of each solution, because consumption was measured in mass. Using the beginning and ending weights, the approximate weight on day 8 of treatment was extrapolated, and the ethanol and corticosterone doses for days 7 and 8 were calculated based on this weight. All rats in this experiment were tested on the EPM on either postnatal day 46 or 47 and on the FST on postnatal day 47 or 87 using the methods described for experiment 1.

#### Collection and measurement of plasma corticosterone

To determine the amount of corticosterone released after the FST, blood was collected by tail nick from each rat immediately, 45 min and 90 min after removal from the test. No baseline was obtained before the FST in order that behavior in the FST would not be affected by the blood sampling. Blood samples were centrifuged at 3,000 rpm and 4°C for 20 min. Plasma was collected and stored at -20°C until corticosterone was measured using enzyme-linked immunosorbent assay kits (Neogen, Lansing, MI, USA). Corticosterone was extracted from the samples using ethyl ether and the samples were reconstituted in a buffer provided in the kit. The assay was run entirely as specified in the instructions for the kit, except that the reconstituted samples were diluted twofold compared to the kit instructions in order for the stress levels of corticosterone to be readable within the standard curve. The minimum detection level for the assay is 1 μg per tube. The antiserum crossreacts with deoxycorticosterone (38%), and only slightly with cortisol (1.1%), testosterone (0.12%), and estradiol (<0.01%). The intra-assay and interassay reliabilities were both less than 10%.

## Results and discussion

### Experiment 1

#### Weight

**Figure 1:**
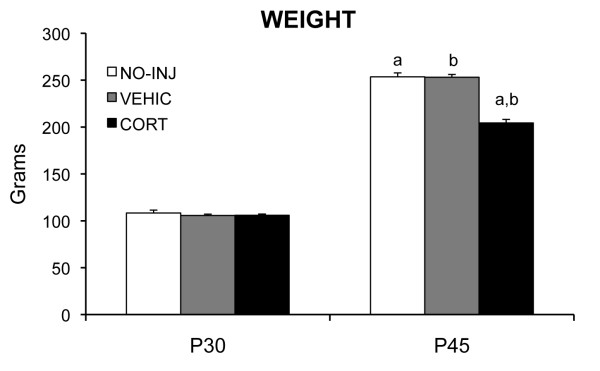
**Mean (SEM) weight at postnatal day 30 (P30) and P45, the first and last day of treatment for the corticosterone injected (CORT), vehicle injected (VEHIC) and no injection (NO-INJ) groups**. Matched letters indicate significant (*P *< 0.05) differences between groups.

There were no group differences in weight on postnatal day 30 (F_1,21 _= 0.02, *P *= 0.93), but by postnatal day 45, corticosterone injected (CORT) rats weighed less than vehicle injected (VEHIC) and no injection (NO-INJ) rats (F_1,21 _= 10.78, *P *= 0.001, see Figure 1). The finding of reduced weight gain of CORT rats is consistent with the evidence of reduced weight gain or weight loss after exogenous corticosterone treatment in adult rats (for example, [28,36]) and after chronic stressors in adolescent and in adult rats (for example, [43-45]).

#### EPM

**Figure 2:**
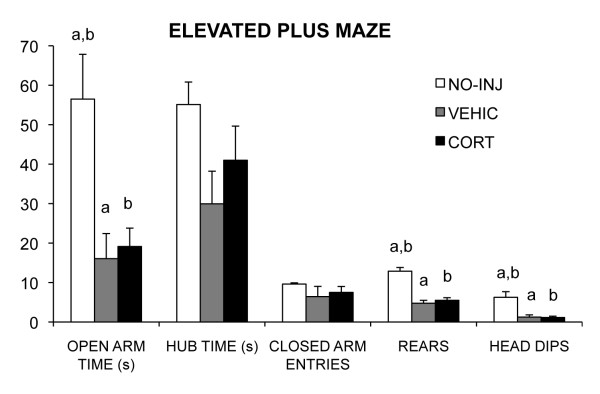
**Mean (SEM) of the behavioral measures for the elevated plus maze (EPM) for corticosterone injected (CORT), vehicle injected (VEHIC) and no injection (NO-INJ) groups**. Matched letters indicate significant (*P *< 0.05) differences between groups within each behavioral measure.

Although vehicle injection had no effect on weight gain, both VEHIC and CORT treated rats had increased anxiety-like behavior compared to NO-INJ rats. NO-INJ rats spent more time on the open arm (F_1,21 _= 7.96, *P *= 0.003, see Figure 2) than did VEHIC (*P *= 0.002) and CORT (*P *= 0.002) rats, which did not differ (*P *= 0.78). The same pattern of significant differences as time in the open arm were obtained for the percentage of time in the open arm using time in open and closed arms without time in the hub as a denominator (F_1,21 _= 7.96, *P *= 0.003, (mean, ± SEM) = VEHIC rats (6.4%, ± 2.6), CORT rats (7.6%, ± 1.6), and NO-INJ rats (23.7%, ± 5.0)). Differences were less marked using percentage of open arm entries relative to total arm entries as the index of anxiety (F_1,21 _= 3.65, *P *= 0.045, with only VEHIC rats having a lower percentage of open arm entries (20.8%, ± 6.8) than NO-INJ rats (37.7%, ± 2.75), and neither group different from CORT rats (29.8%, ± 1.9)). NO-INJ rats reared and head dipped more than did VEHIC and CORT rats, which did not differ (F_1,21 _= 32.45, *P *< 0.0001, F_1,21 _= 10.41, *P *< 0.0001, F_1,21 _= 4.76, *P *= 0.02, see Figure 2). The differences among the groups in time spent in the hub of the EPM and the number of closed arm entries were not significant (F_1,21 _= 2.72, *P *= 0.09 and F_1,21 _= 3.11, *P *= 0.07).

These results suggest that stress of injection is sufficient in adolescence to increase anxiety-like behavior. Although acute injections are known to be a mild stressor (for example, [46]), adult rodents readily habituate to repeated injections (for example, [47,48]). Further, in studies of adults, even when a higher number of injections were used than the 16 used here, differences are observed between vehicle-treated and corticosterone-treated groups [28,29,36,49]. Thus, adolescents may be more sensitive to injection stress than are adults.

#### FST

**Figure 3:**
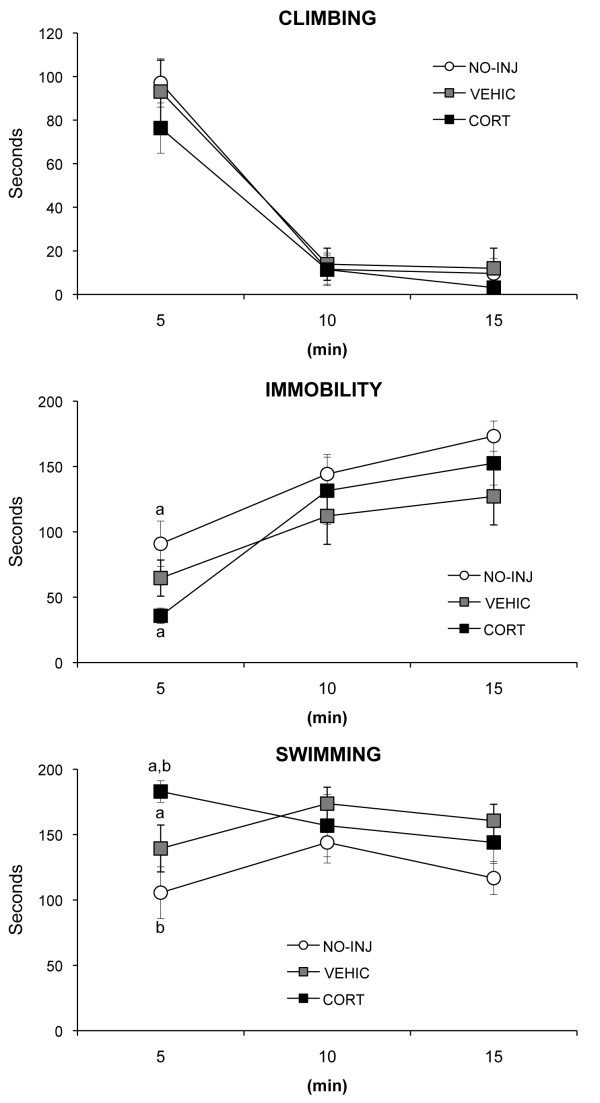
**Mean (SEM) of the behavioral measures for the forced swim test (FST) for corticosterone injected (CORT), vehicle injected (VEHIC) and no injection (NO-INJ) groups**. Matched letters indicate significant (*P *< 0.05) differences between groups within each behavioral measure.

There was no main effect or interaction of group and time on climbing or immobility (all *P *> 0.19) (see Figure 3). The interaction of group and time on swimming approached significance (F_4,42 _= 2.48, *P *= 0.06). The three groups did not differ in latency to immobility (*P *= 0.72, data not shown). To explore the possibility that group differences were masked by low power by inclusion of several timepoints in the analysis (shorter times than 15 min have been used for analysis by others, for example [29]), a multivariate analysis of variance (MANOVA) was computed for climbing, immobility, and swimming, using only the first 5 min in the FST. The multivariate test of group was significant (Pillai's trace F_6,40 _= 2.73, *P *= 0.025), with significant between subject effects for immobility (F_2,21 _= 4.32, *P *= 0.027) and for swimming (F_2,21 _= 6.78, *P *= 0.005). Post hoc analyses indicated that CORT rats spent less time immobile and more time swimming than did NO-INJ rats and more time swimming than VEHIC rats (all *P *< 0.05) (see Figure 3).

Thus, the effects of treatment were limited to the first 5 min of the test and were in the opposite direction than predicted. The measure of depressive behavior in the FST is immobility, and thus CORT rats showed less depressive behavior than did NO-INJ rats. The VEHIC rats' behavior fell between that of the CORT and NO-INJ rats. An alternative explanation is that the increased swimming and decreased immobility of CORT rats is a reflection of their increased anxiety evident in the EPM. Anxiety symptoms are as common as depressed mood is among people meeting the criteria for major depressive disorder [50], and there is evidence that anxiety may be a precursor for the development of depression [51]. Nevertheless, there is increasing evidence of both shared and distinct neural mechanisms underlying anxiety and depressive behaviors (for example, [52]).

Others also have reported the apparent contradiction of increased anxiety and decreased depressive behaviors in animal models. For example, knockout (KO) mice for the 5-hydroxytryptamine (5HT)_1A _receptor also exhibited increased anxiety in an open field test and increased swimming and decreased immobility in the FST [53]. The authors interpreted the behavior in the FST as further evidence of increased anxiety and emotional reactivity rather than to decreased depression, and they suggested the differences in KO mice might involve increased serotonergic turnover. More recently, KO mice of the 5HT_1A _autoreceptor had increased anxiety and did not differ from controls in the FST, whereas KO mice of the 5HT_1A _heteroreceptor had increased immobility in the FST and did not differ in anxiety from controls [52]. In adults, chronic injection of corticosterone decreases neural responsiveness to serotonin (for example, [54]) and increases depressive behavior in the FST (for example, [28,29,36]). One possibility for the difference in the results of the previous studies in adults whereby injection of corticosterone increased depressive behavior compared to our finding of reduced depressive behavior (or increased anxiety) for adolescents is that our study involved fewer injections than those in studies of adults (16 versus 21 injections). A stronger possibility is that the differences between the studies of adults and ours with adolescents are because of developmental differences in susceptibility to the effects of stress/corticosterone. Studies of adults have found more robust differences between vehicle injected and corticosterone injected groups, thus adults may be less susceptible to injection stress than are adolescents. The ongoing maturation of neural circuitry underlying emotional behavior, which is evident, for example, in the different responses of adolescent and adult rats to selective serotonin reuptake inhibitors (for example, [55]), may render the circuitry more vulnerable.

In experiment 2, we eliminated the possibility of stress of injection by administering corticosterone in the drinking water. The method, which involves dissolving corticosterone in ethanol, was based on established procedures that have effectively mimicked many of the effects of chronic stress in adult rats (for example, [31-33]). Because the comparable studies available were conducted only in adults, we were concerned that ethanol alone may have an effect in adolescents. Thus, two control groups were used, one group was given 2.5% ethanol to drink and the other was given water only. To better assess the specificity of effects to developmental stage, an additional three experimental groups received treatment in adulthood.

### Experiment 2

#### Weight

**Figure 4:**
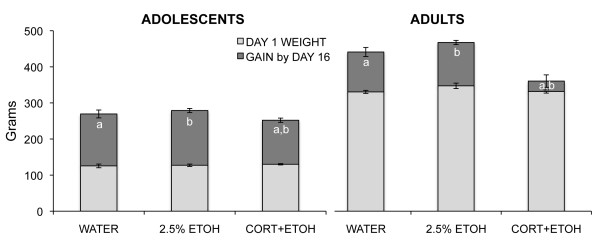
**Mean (SEM) weight at postnatal day 45 the last day of treatment for rats ingesting corticosterone dissolved in 2.5% ethanol (CORT-ETOH), 2.5% ethanol (ETOH), or water (WATER)**. The lighter colored portion of the column represents weight at postnatal day 30, the first day of treatment, and the darker colored portion of the column represents weight gain during the 16 days of treatment. Matched letters indicate significant (*P *< 0.05) differences between groups in weight gain for adolescent and for adult groups.

There were no group differences in weight on postnatal day 30 (F_1,17 _= 0.54, *P *= 0.93), but by the 16th day of treatment on postnatal day 45, CORT-ETOH rats had gained less weight than ETOH and WATER rats, which did not differ (F_1,17 _= 4.37, *P *= 0.029, see Figure 4). There were no weight differences for the adult groups on the first day of treatment (F_1,17 _= 2.84, *P *= 0.09), but by the 16th day of treatment, CORT-ETOH rats had gained less weight than ETOH and WATER rats, which did not differ (F_1,17 _= 13.85, *P *< 0.0001, see Figure 4). Thus, the effects of corticosterone treatment in the drinking water were similar to the results in other studies (for example, [31,56]) and to the effect of corticosterone injection in experiment 1, and the effects were comparable for adolescents and adults. No effect of 2.5% ethanol was observed on weight.

#### Intake

**Figure 5:**
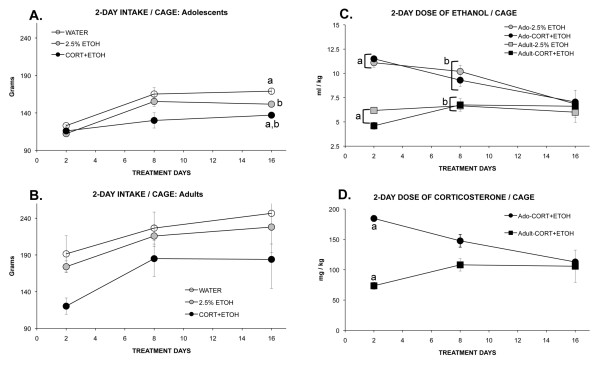
**Mean (SEM) intake of fluid in grams in 2 days per pair of rats during 16 days of treatment for rats ingesting corticosterone dissolved in 2.5% ethanol (CORT-ETOH), 2.5% ethanol (ETOH), or water (WATER) as adolescents (a) or as adults (b)**. **(c) **Shows the dose of ethanol consumed by body weight and **(d) **provides the dose of corticosterone consumed by body weight. Matched letters indicate significant (*P *< 0.05) differences between groups on a treatment day. The main effect of treatment day was significant in (b), in (c) for adolescents only, and in (d) for both adolescents and adults, although in opposite directions (all *P *< 0.05).

Treatment altered the intake of the three groups differently for adolescents than for adults. For adolescents, there was an interaction of treatment day and group (F_2,14 _= 3.25, *P *= 0.04): The intake of CORT-ETOH rats was less than that of ETOH rats (*P *= 0.01) and less than that of WATER rats on day 16 of treatment (*P *< 0.0001) (see Figure 5a). For adults, although the intake of CORT-ETOH rats was less than that of the other groups, only the effect of treatment day was significant (F_2,14 _= 6.42, *P *= 0.01), whereby intake increased over days (see Figure 5b). Thus for adults, there seemed to be an immediate aversion to corticosterone that reduced intake throughout, whereas in adolescents, corticosterone did not reduce drinking initially. The reduced drinking by day 16 of CORT-ETOH adolescent rats may be related to their reduced body weight or to an aversion that was slower to develop. For neither age group was the intake of rats drinking 2.5% ethanol different from those rats drinking water only.

The interaction of treatment day and age was significant for the dose of ethanol consumed (ml/kg) (F_2,14 _= 15.99, *P *< 0.0001): For adolescents, the dose of ethanol consumed (ml/kg) decreased over treatment days (F_2,10 _= 84.95, *P *< 0.0001) similarly for the CORT-ETOH and ETOH rats (see Figure 5c). For adults, there was no effect of, or interaction of, treatment day and group. The higher dose of ethanol ingested by adolescents than by adults was significant only at treatment day 2 and 8 (both *P *< 0.0001). The interaction of age × treatment day was significant for the dose of corticosterone (mg/kg) consumed by the CORT-ETOH groups (F_2,14 _= 6.42, *P *= 0.01): For adolescents, the dose of corticosterone (mg/kg) consumed by the CORT-ETOH rats declined over time (F_2,6 _= 309.3, *P *< 0.0001), and for adults (F_2,6 _= 91.5, *P *< 0.0001), the dose of corticosterone (mg/kg) consumed increased over time (see Figure 5d). The higher dose of corticosterone ingested by adolescents than by adults was significant only at treatment day 2 (*P *= 0.02). These results highlight the difficulty in administering comparable dosages in drinking water to rats of different stages of development.

#### EPM and FST

**Figure 6:**
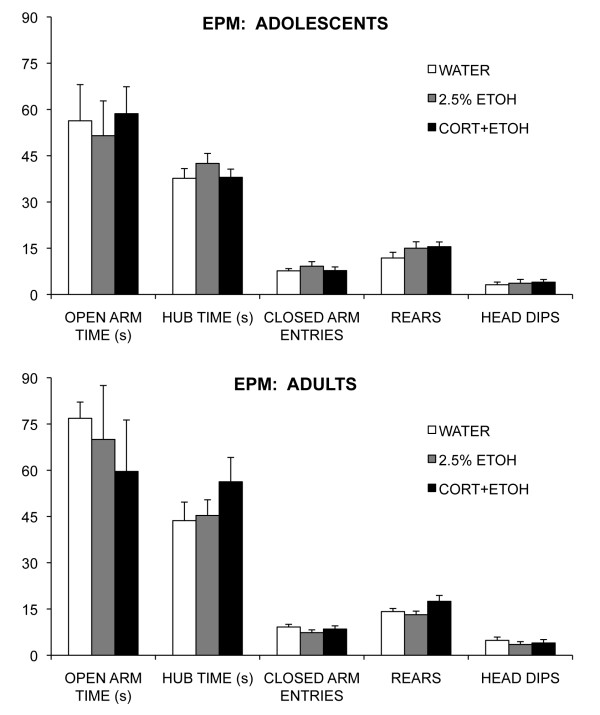
**Mean (SEM) of the behavioral measures for the elevated plus maze (EPM) for rats ingesting corticosterone dissolved in 2.5% ethanol (CORT-ETOH), 2.5% ethanol (ETOH), or water (WATER) as adolescents (upper panel) or as adults (lower panel)**. Matched letters indicate significant (*P *< 0.05) differences between treatment groups for each behavioral measure within each age group.

**Figure 7:**
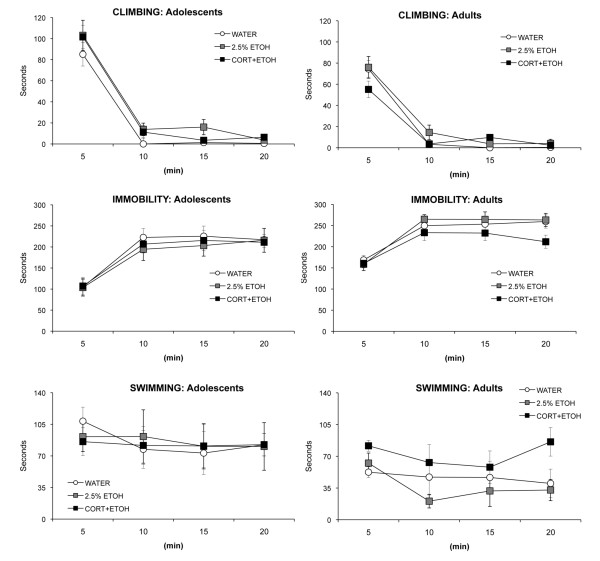
**Mean (SEM) of the behavioral measures for the behavioral measures for the forced swim test (FST) for rats ingesting corticosterone dissolved in 2.5% ethanol (CORT-ETOH), 2.5% ethanol (ETOH), or water (WATER) as adolescents (left panels) or as adults (right panels)**.

There were no differences among the groups for any of the EPM measures for either adolescents (all *P *> 0.32) or adults (all *P *> 0.13). Adolescents spent less time in the center hub than did adults, but no other age difference was significant (F_1,34 _= 4.26, *P *= 0.047) (see Figure 6). Although there was effect of time for the FST measures, with climbing and swimming decreasing and immobility increasing with time for both age groups (see Figure 7), the only effect of group was a group × time interaction (F_6,51 _= 2.63, *P *= 0.027) for time spent climbing by adults. No group comparison, however, was significant at any timepoint.

There are several possibilities for the lack of an effect of corticosterone treatment on anxiety-like and depressive behavior when administered in the drinking water compared to when injected. One possibility is that we would have uncovered behavioral differences among the groups using a broader range of tests of anxiety-like and depressive behavior. Another possibility is that, at least in adolescence, corticosterone is not as effective at altering emotional behavior in the absence of actual stressful experiences, such as injection. Of note, in experiment 1, the effect of injection of corticosterone on behavior was only marginally greater than that of injection of vehicle. Exogenous corticosterone and chronic stress do not always have the same effects when directly compared. Although both 21 days of 400 μg/ml corticosterone in drinking water and 21 days of chronic stress (restraint 6 h/day) caused retraction of hippocampal dendrites, only chronic stress impaired spatial memory [57], and the two manipulations differed in their effects on fear conditioning [33] and had the opposite effects on proteins involved in synaptic plasticity in the piriform cortex [58]. Whereas 21 days of injection of corticosterone decreased the number of cells expressing reelin in the hippocampus, 21 days of daily restraint did not [59]. Nevertheless, the differential effects of chronic stress versus exogenous administration of corticosterone do not explain the absence of an effect of corticosterone observed in experiment 2.

Another possibility is that 16 days is insufficient to have an effect with 400 μg/ml in the drinking water, especially when the pharmacokinetics of this method of delivery differs from a bolus injection, which is more similar to that produced by a bout of restraint. Consistent with the possibility that dose may be a factor is that injections of 10 mg/kg corticosterone in adult rats are sufficient to cause dendritic retraction in the hippocampus (for example, [60,61]) but higher doses are required to increase depressive behavior in the FST [28]. Lastly, the evidence for an effect of corticosterone delivered in the drinking water on anxiety and depressive behavior is not as substantial as it is for corticosterone delivered by injection. The available studies used mice rather than rats and lower doses (<50 μg/ml) and found reduced depressive behavior when provided for 1 to 4 days [62], increased depressive behavior when provided for 14 days and tested 2 weeks later [63], but did not affect depressive behavior in the FST when provided for 4 weeks [64]. Thus, given the advantages of administering corticosterone in the drinking water (for example, no handling or injection of the animals is involved), it would be worthwhile to test the effects of the 400 μg/ml dose provided over a longer time frame on behavior.

#### Plasma corticosterone

**Figure 8:**
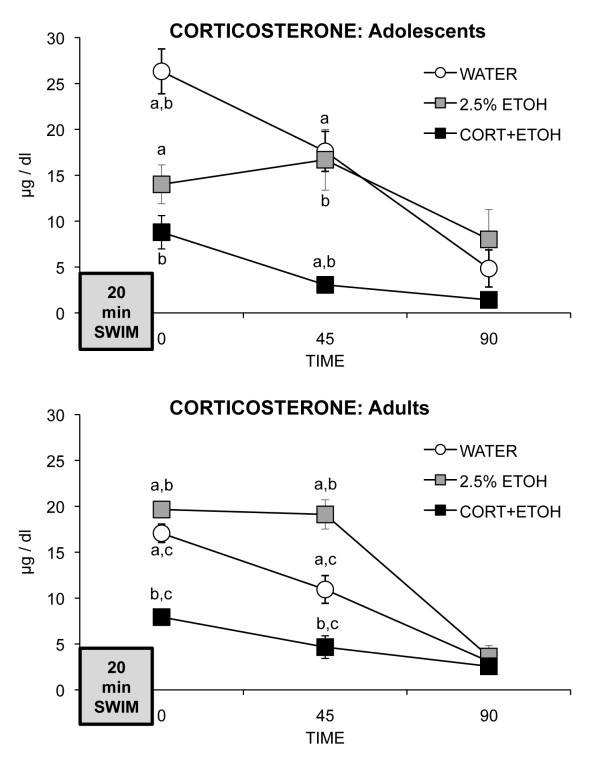
**Mean (SEM) concentrations of corticosterone at timepoints after removal from the forced swim test (FST) for rats ingesting corticosterone dissolved in 2.5% ethanol (CORT-ETOH), 2.5% ethanol (ETOH), or water (WATER) as adolescents (upper panel) or as adults (lower panel)**. Matched letters indicate significant (*P *< 0.05) differences between treatment groups at a timepoint within each age group.

Baseline samples were not collected before the FST to prevent interference with subsequent behavior. Plasma concentrations of corticosterone obtained at timepoints after the FST indicated that both corticosterone treatment and the 2.5% ethanol vehicle altered HPA function. For adolescents, the interaction of sampling time and group was significant (F_4,34 _= 5.73, *P *= 0.001) (see Figure 8). At removal from the FST and 45 min later, CORT-ETOH rats had lower corticosterone concentrations than had WATER rats (both *P *< 0.0001). ETOH rats had lower corticosterone concentrations than had WATER rats only immediately after removal from the FST (*P *= 0.001). Group differences were not significant 90 min after the FST.

For adults, the interaction of sampling time and group was significant (F_4,34 _= 16.55, *P *< 0.0001) (see Figure 8). On removal from the FST and 45 min later, CORT-ETOH rats had lower corticosterone concentrations than had ETOH (both *P *< 0.0001) and WATER rats (both *P *< 0.01), and ETOH rats had higher corticosterone concentrations than had WATER rats (both *P *< 0.05). The groups did not differ 90 min after the FST.

Reduced adrenal function after lengthy exogenous treatment with high doses of corticosterone is a typical finding, because of the reduction in size of the adrenal gland caused by exogenous corticosterone, which is in contrast to the increase in size of the adrenal produced by chronic stress (for example, [28,31]). Thus, the lack of effect of treatment on behavior cannot be attributed to a lack of delivery of the treatment. The effect of 2.5% ethanol vehicle on corticosterone release was unexpected, as most studies of corticosterone in the drinking water have not included a 'no 2.5% ethanol' control group (for example, [33,56,58,65]). In adolescents, the low dose of ethanol in the drinking water reduced corticosterone release after the FST, but not to the same extent that corticosterone and ethanol combined did. In adults, ethanol prolonged corticosterone release after the FST. Whether the effect of ethanol involved effects on the adrenal gland or at a higher level of control of the HPA axis in either adolescents or adults is unknown. After acute or chronic ethanol exposure to adult rodents, usually heightened HPA function is reported, with changes evident at all levels of the HPA axis (such as increased adrenal weight/corticosterone content, higher expression of pituitary pro-opiomelanocortin (POMC) mRNA, of hypothalamic corticotropin-releasing hormone (CRH) mRNA) that are consistent with higher stress-induced corticosterone release (for example, [66-70] although for evidence of dampened HPA function after alcohol exposure, see [71]).

The differences in the effects of ethanol in the two age groups may reflect differences in their developmental stage (for example, [72]) or that the dose of ethanol consumed was higher for adolescents than for adults. In adults, although activation of the HPA axis was found to a first dose of alcohol, self-administration or experimenter administration of alcohol was found to dampen HPA responses to subsequent stressors up to 24 days after removal of the alcohol (reviewed in [71]). One study of adolescent exposure to ethanol (alcohol vapors) found evidence of reduced HPA function when tested in adults [73]. In contrast, binge drinking (3 g/kg/day) beginning at postnatal day 37 increased the expression of secretagogues of ACTH in the paraventricular nucleus of the hypothalamus in adolescence [74]. Such studies, however, involved much higher doses than used here (for example, a bolus injection of 4.5 mg/kg to a rat is far more than the 200 mg/kg highest dose consumed by a pair of rats over a 48 h period in the present study). Nevertheless, the present results suggest that a no vehicle control group should be used in future studies using ethanol as a vehicle for corticosterone.

## Conclusions

Administration of exogenous corticosterone has proven to be an effective preclinical model of depression in adult rodents to study the relationships among stress, glucocorticoids, and depression (reviewed in [14]). The findings of the present experiments suggest there are challenges to overcome in the use of exogenous corticosterone to investigate the heightened vulnerability of adolescents to stressors. First, the stress of injection masks potential effects of exogenous corticosterone in adolescents, whereas robust differences between vehicle-injected and corticosterone-injected subjects are found routinely in adults [14]. Second, corticosterone delivered in the drinking water does not allow for sufficient control of dosing for comparable treatment in adolescents and adults. Administration procedures that do not involve ethanol, such as the use of corticosterone hemisuccinate rather than corticosterone, are recommended, because even the low dose of ethanol used here affected corticosterone release in both adolescents and adults. Further, although the dose of corticosterone used (400 μg/ml) represents the high end of doses administered in such a way, 16 days of treatment did not produce effects on behavior in the EPM and the FST, although many studies have found chronic stress in adolescence to affect behavior in the EPM and the FST within shorter timeframes (reviewed in [13]). Thus, although this route of administration overcomes the problem of stress of injection, the more sustained pattern of corticosterone exposure through such oral administration may not allow for marked effects on mood-related behavior to be observed in rats. In sum, the present results highlight challenges to overcome in the use of exogenous corticosterone treatments in animal models to study sensitivity to stress and mood disorders in adolescence.

Clinicians have long recognized there are unique features to mood disorders in adolescence compared to in adulthood, irrespective of whether or not a mood disorder is the same fundamental disease at each age (for example, [75]). In humans, anxiety is primarily a disorder of childhood and adolescence, with anxiety in adults stemming from adolescence, and stress is a risk factor for anxiety in adolescence [76,77]. Immaturity of the relevant regulatory mechanisms in adolescence is thought to underlie the difference in performance on behavioral tests of anxiety of adolescent and adult rats [78,79]. In experiment 1, injected and corticosterone-treated adolescents had increased anxiety-like behavior compared to control rats, with little difference observed in depressive behavior. Similar treatments in adult rats found greater increases in depressive behavior than in anxiety [14]. These results suggest there are age differences in the behavioral expression of exposure to corticosterone that may parallel the developmental stage-specific manifestations of mood disorders in humans. In humans, the age of onset for depression is later than for anxiety, which also suggests that the maturation of relevant neural circuitries may determine which mood disorder or symptom is evident at each age. These points underscore the relevance of animal models for understanding the pathogenesis of mood disorders and the importance of investigating the range of developmental stages in both preclinical and clinical studies.

## Competing interests

The authors declare that they have no competing interests.

## Authors' contributions

PW and CMM contributed equally to the design, analysis, and writing of the manuscript. PW implemented the research and collected the data. Both authors read and approved the final manuscript.

## Authors' information

Patti Waters, MA, Department of Psychology, Brock University, 500 Glenridge Avenue, St Catharines, Ontario, L2S 3A1, Canada. Cheryl M McCormick, PhD, Canada Research Chair in Behavioural Neuroscience, Professor, Centre for Neuroscience and Department of Psychology, Brock University 500 Glenridge Avenue, St Catharines, Ontario, L2S 3A1, Canada.
